# Physicians as medical tourism facilitators in Nigeria: ethical issues of the practice

**DOI:** 10.3325/cmj.2016.57.601

**Published:** 2016-12

**Authors:** Olusesan Ayodeji Makinde

**Affiliations:** 1 Viable Knowledge Masters, Abuja, Nigeria; 2 Demography and Population Studies Programme, Schools of Public Health and Social Sciences, University of the Witwatersrand, Johannesburg, South Africa sesmak@gmail.com

Globalization through the internet provides an opportunity to market services, including health care, across the world. Marketing health care services over the internet and social media is an increasing practice carried out by medical tourism facilitators (MTFs) so as to reach a wider audience (1). Traveling from one country to another to seek health care services has been termed medical tourism. In addition to marketing health care services, MTFs mediate between medical tourists and potential caregivers at medical tourist destinations (2). The internet remains the medium by which potential medical tourists learn about the services offered at possible medical tourist destinations several miles away. Occasionally, advertisements by MTFs contravene the laws of their target countries. An earlier paper from Nigeria identified that the code of medical ethics of the country did not allow in-country medical practitioners to advertise the services rendered at their practices (3). However, this code of ethics was regularly violated by MTFs marketing health care services over the internet and social media.

Medical tourism is thriving in some low- and middle-income countries, which serve as destination countries for medical tourists. Particularly, India, Thailand, Malaysia, South Africa, and Turkey are among the world’s most prominent medical tourist destinations (4-6). The growth of medical tourism has been a result of the cost of service and lengthy waiting times in developed countries and the absence of specialist services, elitist ideas, and loss of faith in the quality of health care services rendered in developing countries (3,7-9). The global medical tourism industry has been estimated to be worth over 100 billion dollars and projected to grow at an annual rate of between 20% and 30% (10).

Nigerians with an estimated annual spending on medical tourism of between one and 20 billion US dollars are undoubtedly major contributors to this industry (11,12). Many Nigerians travel to countries such as India and the UK for various treatments including cardiac surgeries, neurosurgeries, cosmetic surgeries, orthopedic surgeries, and renal transplant surgeries (13,14). In 2005, the wife of a serving Nigerian president died after undergoing cosmetic surgery in Spain (15). The current president of the country traveled to the UK in June 2016 in order to attend to “a persistent ear infection” (16). This was despite the availability of over 250 ear, nose, and throat specialists and a national ear hospital in the country (16). Several other prominent Nigerians have reportedly received health care services in various countries and for various ailments.

While medical tourism could provide access to health care services that are not available in departure countries, several issues such as cost of service, follow-up after surgery, quality of care, and adverse outcomes are challenges that have plagued the industry (2,13). Not all services provided to medical tourists are of the quality advertised. About 25% of medical tourists who presented in an Indian hospital for care regretted seeking care at this health facility and were unlikely to recommend the practice to their peers (14). Some of the services that medical tourists seek are not ethically allowed in their home countries where they will return upon completion of a procedure, thereby creating a follow-up conundrum. In addition, the risks associated with services are toned down or never mentioned to the medical tourists by MTFs (2). Such observations are not particular to Nigeria as a source country. A study recently reported that nine (39%) of 23 patients who presented at a health facility in Nigeria after receiving neurosurgical care outside the country died from complications of the procedures they had undergone. Upon return to the country, over a quarter of these patients presented with infections necessitating follow-up care that was not initially planned (13) and that incurred unplanned expenses, which pushed the cost of care to astronomical levels. Medical tourists, in their bid to seek care, are exposed to infectious microbes that are uncommon in their native environments, thereby facilitating the transfer of these infectious agents across geographic boundaries (17). Nosocomial infections they acquired abroad can cause devastating outbreaks in their home countries where inherent immunity to such microbes is non-existent.

Patients with medical conditions that are treatable in Nigeria are being referred abroad because of better information on availability of services over the internet and social media, whereas in-country health facilities are held back by the code of medical ethics, which prohibits physician advertisements (18). A report highlighting a young lady who lost her life in India where she had gone to seek orthopedic care that could have been provided locally was recently published (19). Though it was noted that the procedure could have been carried out in the country, it is possible that the late patient and her family were unaware of it because of limited information and the absence of advertising opportunities by the local health practices.

The wording of the code of medical ethics as understood by the author suggests that alongside physicians who are restricted from advertising their services, health practices are similarly restricted, although an Assistant Registrar of the Medical and Dental Council of Nigeria stated in his public lecture delivered in 2015 that, unlike individual physicians, health facilities were not barred from advertising their services.

Recognizing the financial viability of Nigeria as a source of medical tourists, MTF agencies have moved into the country to have direct access to the population. The trend now also includes physicians, who are approached with a request to refer patients to specific practices outside the country and in return receive some financial inducements (20). Little has been documented about physicians acting as MTFs or their agents around the world. While referrals to more advanced centers have been a worldwide practice of service delivery, international referrals and patient recruitments are increasing with globalization and might be open to abuse. Thus, we examined the ethical implications of this practice from a critical point of view.

## Consequences of Physicians Acting as MTFs

The role of physicians in guiding clients to make the best decisions for their care is a long-held practice. However, knowledge asymmetry has been exploited by physicians to increase their financial gain in several situations, which have led to increased rates of cesarean section births and other unnecessary procedures consequent to information imbalance (21,22). Supplier-induced demand (SID), which results from this knowledge asymmetry, has been well-studied for decades. Changes in policies that favor a particular system have been observed to concomitantly lead to a shift and increased delivery of the services promoted by the physicians who offer these services to the patients (21,23). The consequences of physicians trying to maximize their profits is of significant concern as medical tourism is growing and physicians are approached to become MTFs (24).

The engagement of physicians in Nigeria to recruit medical tourists raises ethical concerns, which could lead to abuse if not addressed. There are concerns that physicians are being given financial inducements based on the number of patients they refer to medical tourism centers in different countries (20). These financial inducements could becloud the judgment of these physicians, who may be referring patients for procedures that are not necessary or that could ordinarily be performed within the country, negatively impacting the health system and causing undue hardship for the medical tourist who has to pay huge bills out of pocket. The model of economic benefit to the referring physician, directly linked to the amount the physician is able to generate for these institutions, is a reason for concern. Besides the economic hardship that such decisions may cause to the patient, there are also other social and medical consequences to which the patients are unduly exposed. This includes the psychological effect on the patient who has to travel to an unknown country for (un)necessary care, the possibility of infections that are not easily susceptible to antibiotics in his/her local environment, and the challenge of follow-up after surgical procedures performed outside the country. Engagement of physicians to refer patients to a specific medical tourist destination is a significant conflict of interest that needs to be appropriately reviewed by the authorities.

The absence of a regulatory framework for medical tourism is a major drawback to the MTF profession, which challenges the norm (2). MTFs view their ethical responsibility to a potential client as being equal to that of the client’s physician (2). Many MTFs interviewed in an earlier study identified the potential conflict of interest between their business objectives and the good of the patient (2). Those interviewed were non-clinicians who were in the business for a financial gain, but who were also concerned about the practice of the growing unregulated profession. The trend of physicians doubling as MTFs, as promoted by MTF agencies, raises a significant conflict of interest. Without appropriate checks, medical tourism will be susceptible to SID. MTFs have been signing liability waivers with their clients in case of adverse outcomes. Should a physician referring a patient wish to do likewise, it would be deemed unethical.

Most physicians in developed countries oppose medical tourism because of the ill effects that may arise as a result of these exposures (2). Likewise, few studies that have been published on the topic in developing countries also antagonize the trend of medical tourism because of negative effects occasionally observed in the patients and the overall effect on the health system (3,13). However, physicians acting as MTFs can be a strategy to get their individual buy-in and thereby alleviate their antagonism through direct engagement and financial inducements. Such a step would constitute a moral corruption of the physicians.

The role of a physician in advising a potential medical tourist can often be complex (25). Notwithstanding, many patients do not seek medical advice before traveling for medical tourism. In Australia, only 40% of medical tourists consulted a physician before embarking on a medical tourism journey (10). When they did, the physicians were sometimes the last point of call after a potential medical tourist had already made plans for care abroad. In such a situation, it would be necessary for the physician to critically appraise the need for travel and, while maintaining the autonomy of the patient, provide advice on the pros and cons of making such a trip. The involvement of physicians as MTFs may help address some issues that have been raised by Crozier and Baylis (25) in having a better perspective on the type of care available to their patients at distant health facilities, which are otherwise unavailable in their home countries. However, the conflict of interest must be appropriately managed.

While medical tourism needs to be better regulated by authorities in departure countries, there is a need for health care providers and health system in these countries to focus on increasing the quality of services provided to clients at their practices. It will help improve their confidence in the health system and foster utilization of locally provided health care services (8).

## Conclusion

Increasing use of the internet and social media for marketing health care services by MTFs and the engagement of physicians as MTFs has created a new ethical challenge in medical tourism departure countries. Having physicians as MTFs is a major moral and ethical issue and the absence of a legal framework makes such a professional relationship open to abuse. There is an urgent need for the regulation of medical tourism agencies and physicians who act as MTFs in Nigeria and elsewhere in order to protect patients from abuse. Proposals for control of this practice may include making it a crime for a physician to refer a patient to a foreign health facility for financial gain and the enactment of legislation making it possible to sue a provider over the quality of care at a referral center. Departure countries also need to invest more resources into their health care systems and develop more skilled human resources in order to discourage medical tourism.
